# 

**Published:** 1900-11

**Authors:** 


### PERSONALS.

Dr. Charles M. Cullen, of Philadelphia, is very fond of a fast horse, and frequently tries his speedy ones on the track.

Dr. W. C. Wooton, Piqua, Ohio, will succeed Dr. Coleman Nockolds at the Grand Rapids Veterinary College.

Dr. Guy M. Richards, of North Yakima, Washington, is proprietor of the Fairview Stock Farm and the owner of some very high-grade Holstein cattle.

Dr. W. W. Martin, of Philadelphia, is now engaged in commercial pursuits in the city of Chicago.

Dr. H. D. Fenimore, of Knoxville, Tenn., will visit California for a period, where he will rest for some months.

Drs. Chrisman and Moore, of the Bureau of Animal Industry, were visitors to Philadelphia early in November. They are now stationed at Washington, D. C.

Dr. C. H. Magill, of Philadelphia, will shortly return from Spokane, Washington, where he has been sojourning in the search for renewed health.

Dr. R. S. Huidekoper, of Washington, was a New York visitor early in October, and the guest of Dr. H. D. Gill for a period.

Dr. H. G. Black, of Wilkesbarre, Pa., has severed his connection with Messrs. Hogg and Phipps, of that city, and located at Atlantic City, N. J.

Dr. W. S. Manley, Pittsburg, Kan., has relinquished his practice for a period of six months and entered the Western Veterinary College of Kansas City, Mo.

Dr. E. T. Handy, of Milwaukee, Wis., has gone on a trip to England to adjust an estate in which his interests are involved.

Dr. M. Jacob, an attaché of the Bureau of Animal Industry, has severed his connection with that Department, and will enter upon private practice at Knoxville, Tenn.

Dr. Leonard Pearson, of Philadelphia, was confined to his bed for several days in the latter part of September from tonsillitis.

Dr. A. J. Savage, of Colorado Springs, Col., since his return from Detroit has passed through a serious attack of typhoid fever, but is now reported as convalescent.

Dr. A. F. Schrieber, of Philadelphia, was a visitor to Quarryville, Pa., in September, directing some special work for the State Livestock Sanitary Board.

Dr. A. G. Kern, of Knoxville, Tenn., has resumed his medical studies at the University of Pennsylvania.

Dr. A. J. Wilson, V.S., of Ontario, was an active worker among those who contributed to the success of the Industrial Exposition at Toronto.

Drs. W. S. Kooker, H. B. Cox, H. D. Martein, James Marshall, of Philadelphia, and W. L. Rhoads, of Lansdowne, were among the road-drivers on parade-day in Philadelphia, September 29, 1900.

Dr. L. E. Mead, of Tunkhannock, Pa., fills the rôle of Coroner for Wyoming County.

Dr. W. E. A. Wyman, of Burlington, has returned to Milwaukee, Wis., where, with restored health, he expects to again resume active practice.

Dr. W. Horace Hoskins, of Philadelphia, was one of the reception committee to escort Mr. William J. Bryan from Wilmington to Philadelphia.

The whole-souled veterinarian, Dr. Ramacciotti, of Omaha, Neb., is caricatured in the city papers as a leader among his people and a famed member of the Aks-ar-ben.

Dr. R. S. Huidekoper, of Media, Pa., will become a resident of Washington, D. C., after November 30, 1900.

Dr. W. F. Walsh, of New York City, has an article in the souvenir number (September issue) of the *Horseshoers' Journal* on "The Diseases, Defects, and Ailments of the Hoof."

Dr. Alexander Plummer, of the United States Army service and formerly located at Walla Walla, Washington, recently sent to the Philippine Islands with his cavalry, is now on a furlough and visiting the States.

